# Assessment of green development strategies for manufacturing industry in China's coastal regions under dual-carbon circular economy using analytic hierarchy process

**DOI:** 10.3389/fpubh.2025.1590653

**Published:** 2025-05-21

**Authors:** Lihong Jiang

**Affiliations:** Chongqing Vocational Institute of Engineering, Chongqing, China

**Keywords:** green development, manufacturing, dual-carbon goals, circular economy, analytic hierarchy process

## Abstract

**Introduction:**

China's coastal manufacturing industry is fundamental to advancing national ecological modernization. Its effective green transition is critical for attaining sustainable development. This study investigates the key elements of this industry's green development, operating within the context of a dual-carbon circular economy, and assesses its current progress.

**Methods:**

A multilayered evaluation framework was developed using the Analytic Hierarchy Process (AHP). This methodology was employed to identify and prioritize crucial factors influencing the green development of the coastal manufacturing industry. Furthermore, the study systematically examined the multidimensional impacts of three principal AHP-derived factors on the industry's quality of green development.

**Results:**

The analysis indicates a consistent improvement in the overall quality of green development within the industry. However, the findings also highlight several significant challenges. Specifically, there is an urgent requirement to bolster the green system, a need to fully leverage the transformative capabilities of green innovation, and a necessity to address the inconsistent pace of green transformation observed across different regions.

**Discussion:**

The steady enhancement in green development quality is a positive trend, yet the identified challenges underscore areas requiring immediate attention and strategic intervention. To expedite the green transformation of the manufacturing sector, it is recommended that enterprises refine their green development strategies by capitalizing on their unique strengths. Simultaneously, government agencies should amplify their policy support and resource allocation to foster and steer this transition toward achieving China's broader ecological and sustainable development objectives.

## 1 Introduction

Sustainable development has become a shared objective of international communities to address global climate change and environmental challenges (1, 2). In this context, dual-carbon goals serve as a key strategy that guides the global economy toward a low-carbon direction to ensure long-term ecological sustainability (3, 4). Recently, numerous scholars have actively explored the topic of high-quality green development under the dual-carbon goals (5–7). They argue that factors such as green credit, governance capacity, and policy environment play a crucial role in driving this transition.

**Figure 1:**
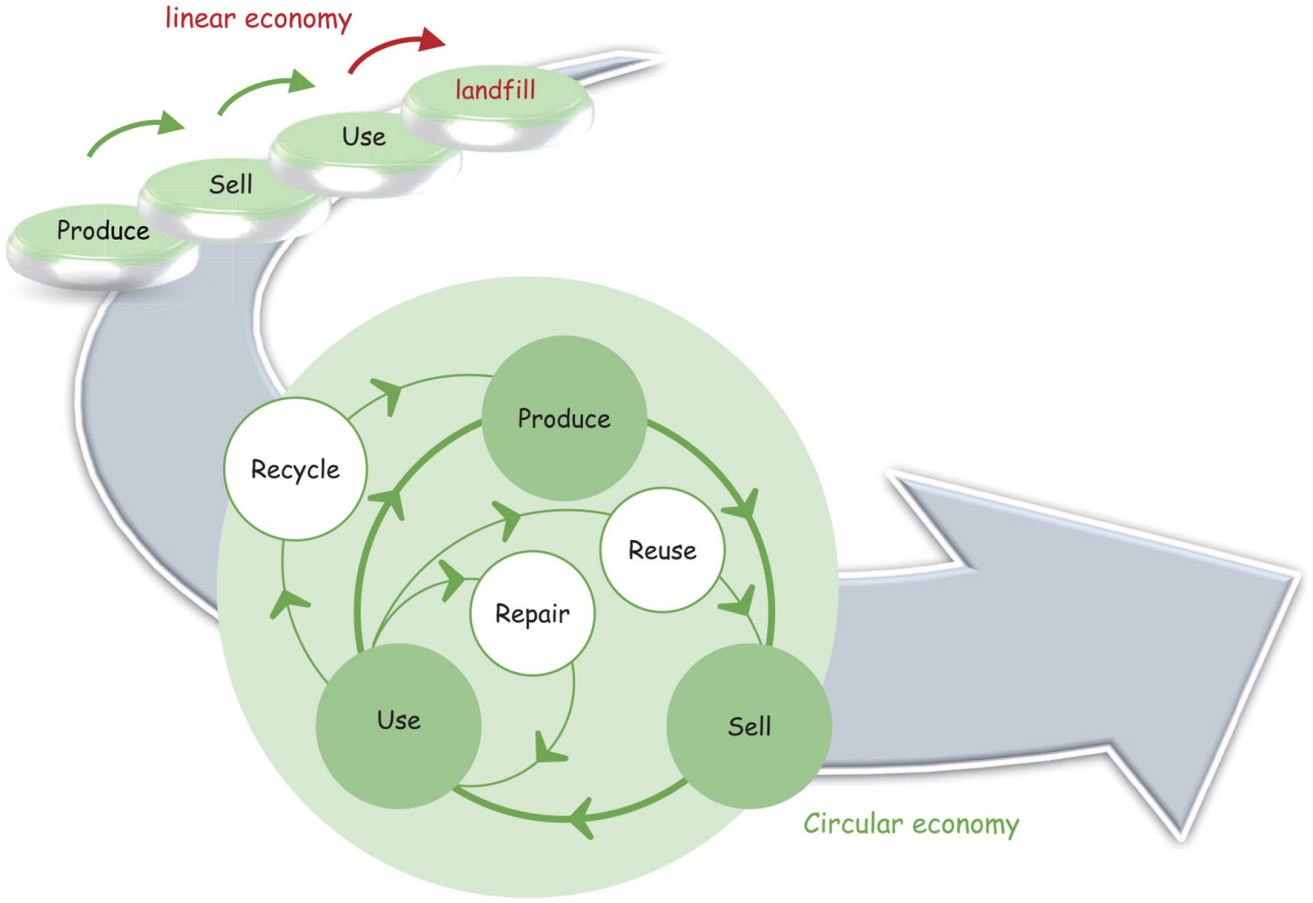
Evolution of the economic development model.

A circular economy has increasingly garnered attention from Chinese scholars as a vital pathway to the dual-carbon goals (8, 9). Unlike the traditional linear model of “Produce-Sell-Use-Landfill,” the circular economy offers a forward-looking framework that emphasizes resource efficiency, waste reduction, and sustainable resource utilization (10). As illustrated in Figure 1, it extends the lifecycle of products, components, and materials, thereby maximizing their utility and economic value (11). The circular economy not only alleviates the environmental pressures of economic activities but also fosters innovation-driven green development through the establishment of a closed-loop system encompassing reuse, recycling, and regeneration. Therefore, this model provides both a practical pathway and a theoretical foundation for achieving high-quality development under the dual-carbon strategy (12).

Coastal regions in China have emerged as the key areas for advancing the dual-carbon goals, benefiting from abundant ecological resources and unique geographical advantages (13, 14). These regions not only bear the responsibility of promoting green transformation but are also instrumental in exploring innovative models of sustainable development. However, transforming ecological capital into a driver of regional development while maintaining a balance between economic growth and carbon reduction remains a major challenge for the regions. Wei et al. (15), using the southeastern coastal region of China as a case study, highlighted the indispensable roles of industrial upgrading and energy decarbonization in the dual-carbon goals. By developing mathematical models to scientifically plan the dual-carbon goals in the coastal regions, Shen et al. (16) provided valuable theoretical insights and practical strategies for addressing the long-standing tension between economic development and carbon emission reduction.

Chinese scholars have extensively studied the green development of the coastal regions within the framework of the dual-carbon circular economy (17, 18). Yet, most research has primarily focused on its macro-level impacts on the green transformation of the coastal regions, with relatively little attention given to the manufacturing industry. The manufacturing industry faces an increasingly urgent need for green transformation as an economic pillar in the coastal regions (19). Traditional manufacturing has long been constrained by high energy consumption, significant pollution, and low resource utilization efficiency, which poses challenges to sustainable development and conflicts with dual-carbon goals (20). Addressing these challenges requires not only technological innovation and policy support but also the establishment of a systematic analytical framework to assess and guide the green transformation of the manufacturing industry.

Building on the above analyses and the widespread application of the Analytic Hierarchy Process (AHP) (21, 22), this paper employs the AHP to systematically evaluate the green development of the manufacturing industry in China's coastal regions under the dual-carbon circular economy. We aim to quantitatively analyze the green transformation process, identify existing challenges, and propose targeted recommendations by constructing a multi-criteria evaluation system. The contribution points of this study are as follows:

1. The AHP is employed to systematically evaluate the green development of the coastal manufacturing industry within the framework of the dual-carbon circular economy, providing valuable theoretical insights into the green transformation of manufacturing.
2. This study offers a comprehensive assessment of the quality of the green transformation in the manufacturing sector across China's coastal regions by integrating kernel density estimation with the Dagum Gini coefficient method. This approach allows for a thorough examination of regional disparities and the identification of key influencing factors.
3. Optimized pathways and strategies for advancing green development in coastal manufacturing are presented, which offer policy recommendations and practical guidance aimed at achieving the dual-carbon goals and fostering sustainable, high-quality economic growth.

This research is organized as follows: Section 1 examines the significance of green development for China's coastal manufacturing industry within the context of the dual-carbon circular economy framework. Section 2 presents the theoretical foundations of the dual-carbon goals, the circular economy, and the concept of green development within the manufacturing industry. Section 3 assesses the progress of green transformation in China's coastal manufacturing industry. Section 4 analyzes the quality of green transformation in the manufacturing industry and examines the causes of regional disparities in green development. Section 5 summarizes the key challenges impeding green development in the manufacturing industry and proposes corresponding recommendations.

## 2 Theoretical basis

### 2.1 Dual-carbon goals

**Figure 2:**
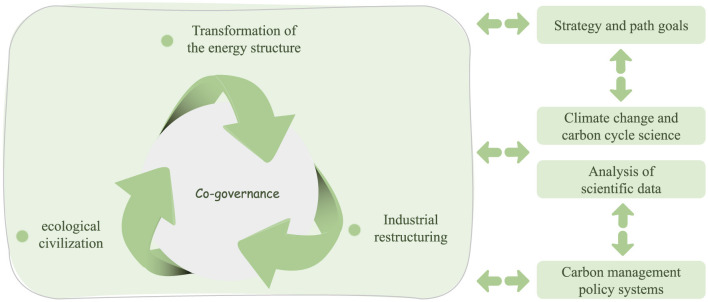
The technological support system for implementing the dual-carbon goals.

Dual-carbon goals represent a major strategic initiative by the Chinese government to address global climate change and promote ecological civilization. Specifically, these goals consist of two-phased targets: achieving carbon peaking by 2030 and attaining carbon neutrality by 2060 (23). Theoretically, the carbon peaking phase emphasizes optimizing the spatial distribution of industrial structures, controlling emission intensity in key sectors, and revolutionizing energy efficiency, thereby guiding total carbon emissions toward an inflection point (24, 25). Building on this foundation, the carbon neutrality phase aims to establish a renewable energy substitution system, commercialize carbon capture technologies, and develop mechanisms to enhance ecological carbon sinks to achieve a dynamic balance between anthropogenic emissions and carbon removal (26). To implement the dual-carbon goals, China has established a multi-level technological innovation system that encompasses basic research, technological breakthroughs, and industrial transformation, with the architectural features of its technology support system illustrated in Figure 2.

### 2.2 Circular economy

The theory of the circular economy was first introduced by American economist Boulding in the 1960s as part of his exploration of ecological economics (27). This theory asserts that economic development should align with environmental carrying capacity and that minimizing resource inputs is essential for achieving energy efficiency and resource conservation (28). Specifically, the circular economy promotes “pollution reduction, resource recycling, and value maximization,” emphasizing the synergistic relationship between technological innovation and ecological, economic, and social systems. Furthermore, in practical applications, it is closely associated with key factors influencing national green economic competitiveness, including environmental protection, high-quality production, and improved living standards.

### 2.3 Definition of green development in the manufacturing industry

The manufacturing industry serves as the cornerstone of the modern industrial system and a key driver of high-quality economic development (29). Under the dual-carbon goals and the circular economy framework, the sector must optimize its production methods by transitioning to a model characterized by “continuously decreasing and recyclable energy consumption, significantly reduced pollutant emissions, progressively lower environmental impact, and markedly enhanced sustainability” (30). This transition aims to establish a development path that integrates economic growth with ecological sustainability, ultimately leading to lower industrial emissions and enhanced green total factor productivity. Therefore, the manufacturing sector must sustain the momentum of green transformation, advancing toward the goals of “low energy consumption, low emissions, high added value, and high productivity” as the foundation for accelerating the development of a strong manufacturing nation.

## 3 Evaluation method

**Figure 3:**
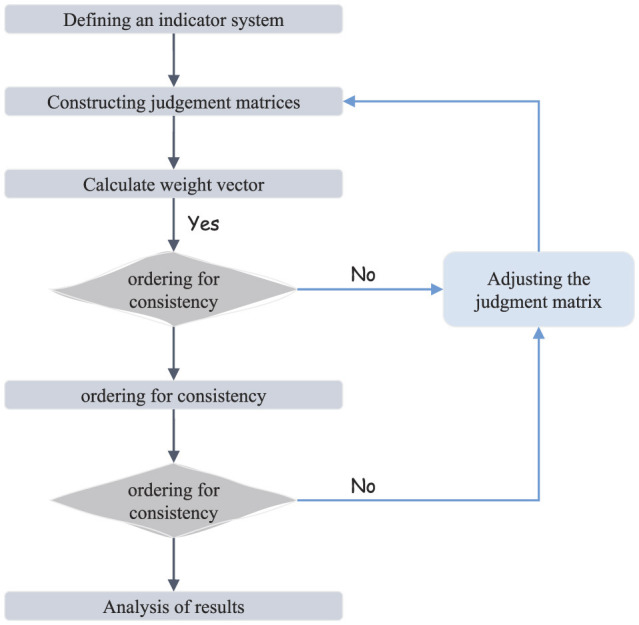
The framework of the AHP model.

To scientifically assess the green development of the manufacturing industry in China's coastal regions within the context of the dual-carbon circular economy, this study employs the Analytic Hierarchy Process (AHP) to develop a structured and objective evaluation model. The AHP modeling process involves three key steps to ensure a systematic and rigorous evaluation. First, a hierarchical structure is established, consisting of three levels: the goal layer, which defines the overall objective of the green development assessment; the criterion layer, which includes major influencing factors such as resource efficiency, environmental impact, and technological innovation; and the sub-criterion layer, which further refines these factors into specific, measurable indicators. Second, a judgment matrix is developed based on expert evaluations, in which pairwise comparisons are performed to determine the relative importance of criteria and sub-criteria. To enhance the reliability of the evaluation, a consistency check is conducted to ensure logical coherence in expert judgments. The weight values of indicators at each level are subsequently computed using eigenvector calculations. Finally, a weighted synthesis algorithm is applied to integrate these weight values, yielding a comprehensive evaluation score for the green development of the manufacturing industry. This systematic approach provides a robust foundation for decision-making, enabling policymakers and industry stakeholders to formulate effective strategies for sustainable development. The detailed AHP modeling process is illustrated in Figure 3.

### 3.1 Constructing criterion system

The green development of the manufacturing industry currently faces three primary challenges: the predominance of high-energy-consuming and high-polluting industries within the industrial structure, a substantial energy efficiency gap compared to developed countries, and the inadequate research and application of ecological environmental protection technologies (31, 32). To address these challenges, we have developed a comprehensive evaluation system for green development, grounded in the principles of scientific rigor, operability, adaptability, comprehensiveness, and real-time performance. This system assesses the green development of the manufacturing industry across three dimensions: resource environment, energy utilization, and green investment, as shown in Table 1.

**Table 1:** Indicators of the criterion system.

**Criterion (*A*_*i*_)**	**Sub-criterion (*A*_*ij*_)**	**Unit**	**Orientation**
Resource environment	Carbon emission intensity per unit of output	t	Negative
	Sulfur dioxide emissions per unit of output	kg	Negative
	Percentage of waste resources reused	%	Positive
	Comprehensive utilization rate of industrial solid waste	%	Positive
Energy utilization	Electricity consumption per unit of output	kWh	Negative
	Growth rate of wind and solar power	%	Positive
	Coal consumption rate for power generation	%	Negative
	Share of renewable electricity consumption	%	Positive
Green investment	Number of green products designed	pcs	Positive
	Number of environmental patents	pcs	Positive
	Number of local environmental regulations	pcs	Positive
	Number of enterprises that have implemented automatic sewage discharge	pcs	Positive

In the resource environment dimension, key indicators include carbon emission intensity per unit of output value, sulfur dioxide emissions per unit of output value, the proportion of revenue from the comprehensive utilization of waste resources, and the industrial solid waste utilization rate. Carbon emission intensity per unit of output value reflects the extent to which the manufacturing industry controls greenhouse gas emissions while achieving economic growth, serving as a crucial measure of low-carbon transition performance. Sulfur dioxide emissions per unit of output value assess the industry's ability to mitigate air pollution and apply clean production technologies. The proportion of revenue from the comprehensive utilization of waste resources indicates the level of resource recycling and reuse, measuring the effectiveness of circular economy practices while also reflecting the economic benefits of resource recovery. The industrial solid waste utilization rate evaluates the effectiveness of technological applications in waste reduction, recycling, and environmentally safe disposal.

For energy utilization, selected indicators include the growth rate of wind and solar power generation, electricity consumption per unit of output value, and the coal consumption rate per unit of electricity generated. The growth rate of wind and solar power generation gauges the industry's transition toward renewable energy, reflecting efforts to reduce dependence on fossil fuels and optimize the energy structure. Electricity consumption per unit of output value directly impacts energy efficiency and serves as a key metric for assessing the effectiveness of energy-saving measures in manufacturing processes, where lower consumption signifies improvements in efficiency. The coal consumption rate per unit of electricity generated measures the cleanliness of energy usage in manufacturing and reflects the role of technological advancements in improving energy efficiency.

In the green investment dimension, indicators include the number of green product designs, the number of environmental protection patents, the number of effective local environmental regulations, and the number of key polluting units implementing automated pollution discharge control. The development and promotion of green products are critical to sustainable manufacturing, as they reflect the degree to which enterprises integrate environmental considerations at the design stage and highlight the industry's level of green innovation. The number of environmental protection patents serves as a key measure of technological progress, with its growth directly indicating enterprise investments in and achievements related to environmental technology research and development. The number of effective local environmental regulations reflects the extent of policy support from local governments in driving green transformation and strengthening the regulatory framework for sustainable manufacturing. Lastly, the number of key polluting units implementing automated pollution discharge control evaluates the application of environmental monitoring and pollution prevention technologies, demonstrating the level of intelligence and precision in corporate environmental management.

In summary, these carefully selected indicators are chosen based on their representativeness and feasibility in assessing the green development level of the manufacturing industry. Collectively, they encompass key aspects of resource utilization, energy efficiency, and environmental investment, offering a comprehensive evaluation of the sector's performance in low-carbon transition, energy conservation, emission reduction, and green innovation.

### 3.2 Calculate index weight value

To ensure the scientific rigor and authority of the evaluation index system, this study employs the Delphi Method for expert consultation (33). Specifically, we conducted a questionnaire survey with 15 experts in relevant fields to obtain the average scores of indicators at both the criterion and sub-criterion layers, based on which we constructed a judgment matrix. To ensure the reliability of the evaluation results, the expert team in this study consists of PhDs or associate professors from economics at universities in the coastal regions of China, including Shandong University of Finance and Economics, Shanghai University of Finance and Economics, Tianjin University of Finance and Economics, Ningbo University, and Hainan University.

In the process of constructing the judgment matrix, we apply the 1–9 scale method proposed by Saaty for quantitative evaluation (34). This method uses numerical values 1, 2, ..., 9 and their reciprocals 1, 1/2, ..., 1/9 as scales to measure the relative importance between hierarchical elements. The scale values reflect the comparative priority of each element, where a value of 1 represents equal importance, and a value of 9 indicates an absolute dominance of one element over another. By conducting pairwise comparisons between elements, an *N*-order judgment matrix is constructed, ensuring the consistency and logical coherence of the evaluation process. The specific scale meanings are shown in Table 2. The score of each indicator reflects its importance, with higher scores indicating greater significance. The relative importance of two indicators can be assessed based on the difference in their scores. A threshold of 0.54 is used to define levels of importance, as outlined in Table 3.

**Table 2:** The 1–9 scale of AHP.

**Scale**	**Description**
1	Equal importance between two elements
3	One element is slightly more important than the other
5	One element is moderately more important than the other
7	One element is strongly more important than the other
9	One element is extremely more important than the other
2, 4, 6, 8	Intermediate values between adjacent levels

**Table 3:** Judgment scale and corresponding value ranges.

**Judgment scale**	**1**	**2**	**3**	**4**	**5**	**6**	**7**	**8**	**9**
*A*_*i*_ − *A*_*j*_	0–0.54	0.54–1.08	1.08–1.62	1.62–2.16	2.16–2.7	2.7–3.24	3.24–3.78	3.78–4.32	4.32–4.86

To verify the consistency of the judgment matrix, we further compute the consistency ratio (*CR*) using the consistency index (*CI*) and the random consistency index (*RI*). The consistency check is crucial to ensure that expert judgments are logically coherent; typically, if *CR* < 0.1, the matrix is considered to have acceptable consistency, allowing the results to be used for further calculations. Based on the expert consultation results, the pairwise judgment matrix **A** at the criterion layer level is expressed as follows:


(1) $$\begin{array}{l} \text{A}=\left[\begin{array}{ccc} A_{11} & A_{12} & A_{13}\\ A_{21} & A_{22} & A_{23}\\ A_{31} & A_{32} & A_{33} \end{array}\right]=\left[\begin{array}{ccc} 1 & 3 & 1\\ 1/3 & 1 & 1/2\\ 1 & 2 & 1 \end{array}\right]. \end{array}$$

Based on the judgment matrix, we use the arithmetic mean method to determine the weight distribution of each indicator. First, the judgment matrix is normalized to eliminate the influence of dimensional differences on the weight calculation. Next, the elements in each column are summed, and the sum of each column is divided by the order *n* of the judgment matrix to obtain the weight vector for each indicator. The specific calculation formula is as follows:


(2) $$\begin{array}{l} w_{i}=\frac{1}{n}\overset{n}{\underset{j=1}{\sum }}\frac{A_{ij}}{\sum_{k=1}^{n}A_{kj}},\left(i=1,2,\ldots ,n\right), \end{array}$$

where *w*_*i*_ represents the weight of the *i*th evaluation criterion. *A*_*ij*_ denotes the element in the *i*th row and *j*th column of the pairwise comparison matrix, indicating the relative importance of criterion *i* compared to criterion *j*. *n* is the total number of criteria. Therefore, the criterion layer weight values $w_{i}={\left[0.443,0.169,0.388\right]}^{\text{T}}$.

To verify the rationality of the judgment matrix, it is necessary to check its consistency. the consistency index (*CI*) can be calculated as follows:


(3) $$\begin{array}{l} CI=\frac{\lambda_{\max}-n}{n-1}, \end{array}$$

where $\lambda_{\max}=\overset{n}{\underset{i=1}{\sum }}\frac{\left[A\bar{w}\right]}{n{\left(w_{i}\right)}_{i}}$ represents the maximum eigenvalue of the judgment matrix. Subsequently, we compute the consistency ratio (*CR*) by:


(4) $$\begin{array}{l} CR=\frac{CI}{RI}, \end{array}$$

where *RI* represents the average random consistency index. Based on the average stochastic consistency test index *RI* index value in Table 4, for the 3rd order matrix, *RI* = 0.58. Therefore, the consistency ratio is *CR* = 0.01. When *CR* < 0.1, the judgment matrix is considered to have an acceptable level of consistency. Similarly, the sub-criterion judgment matrix can be constructed and subjected to a consistency test. Finally, the weight results for both the criterion layer and the sub-criterion layer are shown in Table 5.

**Table 4:** Mean stochastic consistency test metric *RI* metric value.

**Order**	**1**	**2**	**3**	**4**	**5**	**6**	**7**	**8**	**9**	**10**	**11**
*RI*	0	0	0.58	0.9	1.12	1.24	1.32	1.41	1.45	1.49	1.51

**Table 5:** Weight values of indicators in the criterion layer and sub-criterion layer.

**Criterion layer**	**Weight value**	**Sub-criterion layer**	**Weight value**
*A*_1_	0.443	*A*_11_	0.068
		*A*_12_	0.252
		*A*_13_	0.099
		*A*_14_	0.024
*A*_2_	0.169	*A*_21_	0.075
		*A*_22_	0.044
		*A*_23_	0.007
		*A*_24_	0.043
*A*_3_	0.388	*A*_31_	0.203
		*A*_32_	0.058
		*A*_33_	0.062
		*A*_34_	0.065

### 3.3 Sensitivity analysis

Sensitivity analysis is an important tool for testing the robustness of the AHP model. By making small adjustments to input parameters (such as weights, scores, etc.), the resulting output (such as priority rankings) is observed to see if significant changes occur.

### 3.4 Evaluation based on statistical data

**Figure 4:**
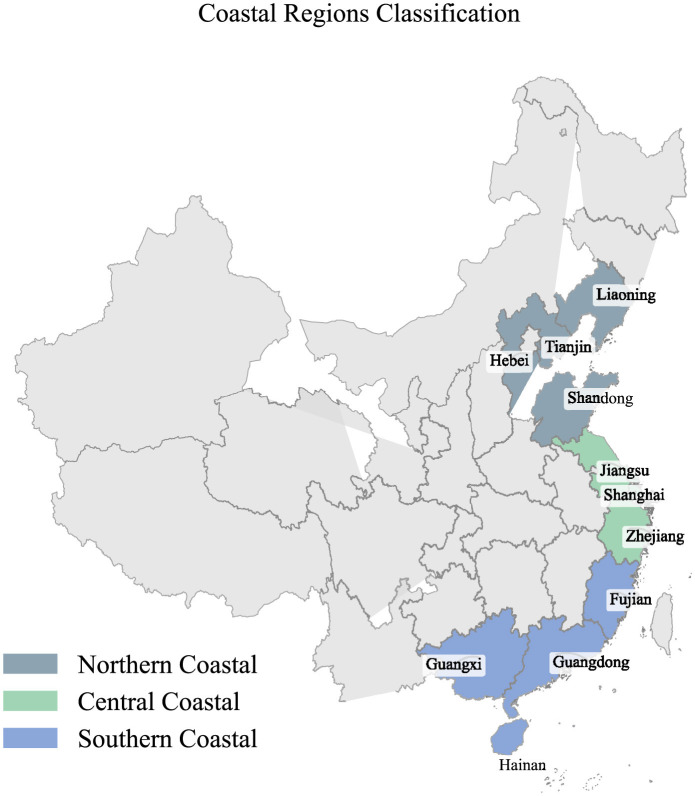
Coastal regions classification.

To objectively assess the green development trends of China's coastal manufacturing industry under the dual-carbon goals, we evaluated the scores for resource environment, energy utilization, and green investment using statistical data from 2017 to 2021 across 12 sub-criteria indicators and their corresponding weight values. Data were obtained from official sources, including the National Energy Administration, the Ministry of Industry and Information Technology of China, the National Bureau of Statistics of China, Wind Information, and CEIC Data. Due to data unavailability for Hong Kong, Macao, and Taiwan, our study focuses on 11 coastal provinces, which are further classified into three regions—Central Coastal, Northern Coastal, and Southern Coastal—based on geographical location for comparative analysis, as shown in Figure 4. Linear imputation is performed using the FORECAST function in Excel to handle missing data. The evaluation process involves two steps. First, the range standardization method is employed to calculate the positive sub-criterion indicators score *S*:


(5) $$\begin{array}{l} S=\frac{V_{i\left(t\right)}-V_{\min\left(t\right)}}{V_{\max\left(t\right)}-V_{\min\left(t\right)}}\times 100, \end{array}$$

where *V*_*i*(*t*)_ represents the value of the *i*-th indicator for the coastal manufacturing industry in year *t*, *V*_min(*t*)_ denotes the minimum value, and *V*_max(*t*)_ the maximum value of the *i*-th indicator in that year. For indicators negatively correlated with the development level, *S* is computed as:


(6) $$\begin{array}{l} S=\frac{V_{\max}\left(t\right)-V_{i}\left(t\right)}{V_{\max}\left(t\right)-V_{\min}\left(t\right)}\times 100. \end{array}$$

**Figure 5:**
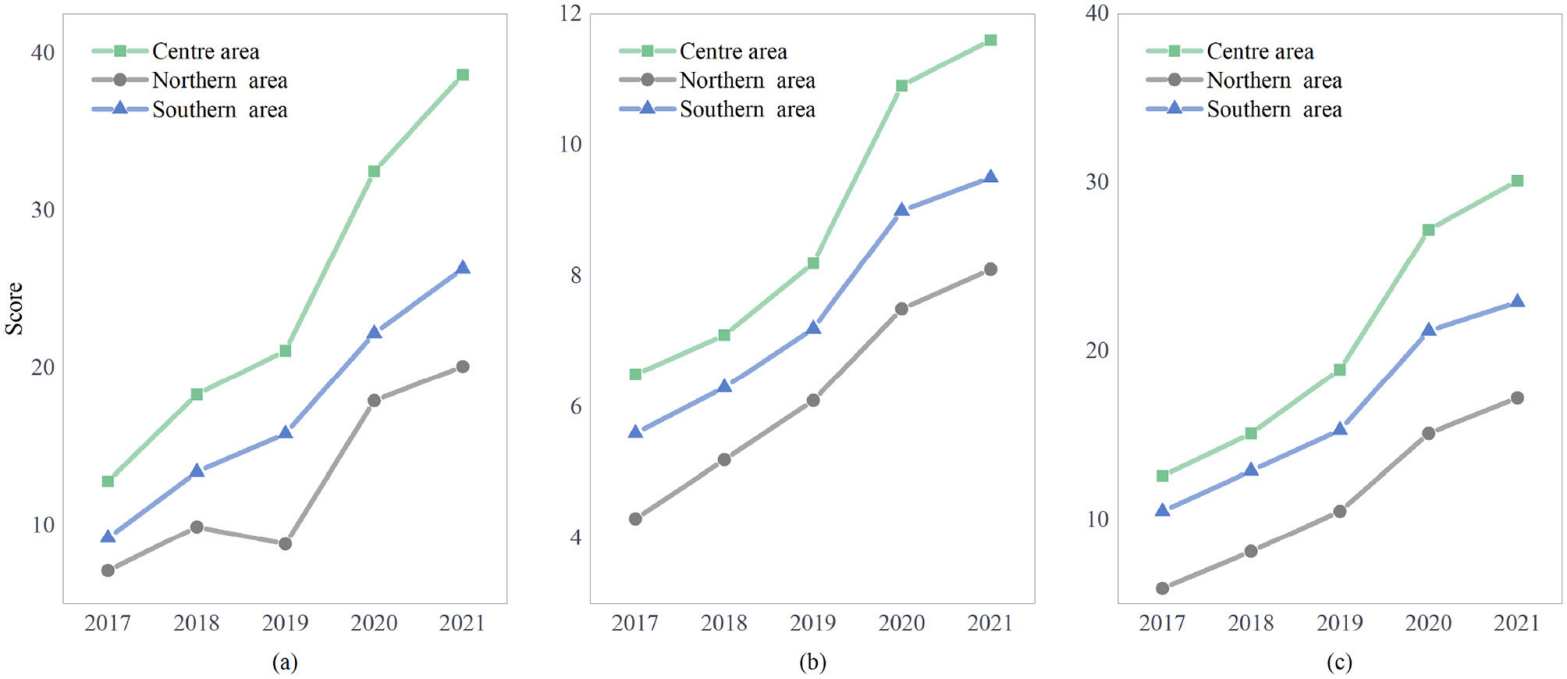
China coastal green development score from 2017 to 2021. **(a)** Resources and environment. **(b)** Energy utilization. **(c)** Green investment.

Second, the score of criterion layer indicators *C* is defined as:


(7) $$\begin{array}{l} C=\sum w_{i}S_{i}, \end{array}$$

where *w*_*i*_ is the weight value of the *i*−th indicator and *S*_*i*_ is its corresponding standardized score. The green development quality scores for China's coastal regions from 2017 to 2021 are illustrated in Figure 5.

### 3.5 Evaluation based on membership function

Based on the membership function method, we conducted a fuzzy comprehensive analysis of the development model of the coastal manufacturing industry under the green dual-carbon circular economy. According to membership function theory, membership degrees are typically classified into three levels: 1, 0.5, and 0. However, when these discrete points cannot accurately describe the membership distribution of the research object, a continuous membership function that reflects actual conditions is selected, and the precise membership degree is determined by identifying elements within a specific interval and applying the corresponding computational formula.

**Figure 6:**
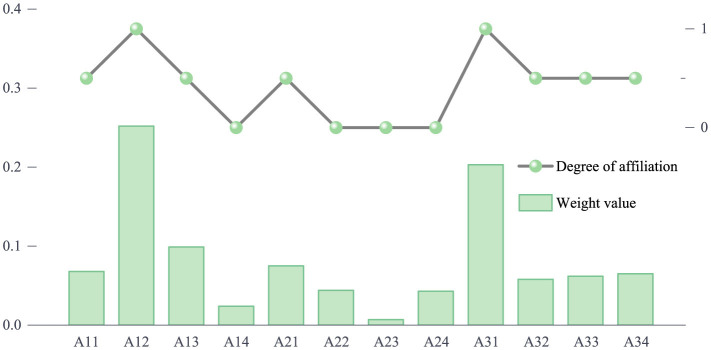
Sub-criterion layer mapping results.

In our study, the weight values at the sub-criterion layer are converted into membership degree values using the membership function method. Based on predefined threshold ranges, these weight values are mapped to 1, 0.5, or 0, as shown in Figure 6. This conversion effectively quantifies the relative importance of each indicator in the green dual-carbon circular economy. For the criterion layer, we further integrate the sub-criterion layer with its corresponding weight values by applying the formula *V* = W^*^A to derive the membership degree values. Accordingly, the membership degree for resources and environment is *V*_1_ = 0.34, for energy utilization is *V*_2_ = 0.04, and for green investment is *V*_1_ = 0.30.

## 4 Analysis of evaluation results

### 4.1 Overall results of green development quality

Figure 5 clearly illustrates the continuous upward trend in the overall green development score of China's coastal manufacturing industry. This growth is primarily driven by the dual-carbon goals and guided by circular economy principles, reshaping the relationship between economic development and environmental sustainability. The improvement in green development can be attributed to a combination of policy measures, economic transformation, and technological advancements. From a policy perspective, the Chinese government has implemented stricter environmental regulations, established a carbon emission trading system, and introduced financial incentives for green manufacturing, all of which have significantly accelerated the green transformation of the manufacturing sector. Additionally, enhanced monitoring mechanisms and accountability systems ensure the effective enforcement of green policies, strengthening environmental governance capabilities. Economically, the acceleration of industrial upgrading in coastal regions, increased investment in green technologies, and rising market demand for environmentally friendly products have further propelled green development. In particular, the shift from traditional high-energy-consuming industries to low-carbon, high-value-added industries has greatly improved resource utilization efficiency. Furthermore, the expansion of green finance, along with the implementation of green credit policies, has provided stable financial support for enterprises undergoing green transformation, further facilitating the transition toward sustainable development. On the technological front, advancements in energy-saving and emission-reduction technologies, the adoption of intelligent manufacturing, and the widespread application of renewable energy have significantly enhanced green development efficiency. Moreover, the integration of digital technologies, such as big data, artificial intelligence, and the Internet of Things (IoT), has enabled intelligent energy management and optimized production processes, further reducing energy consumption and carbon emissions.

Despite these achievements, China's coastal manufacturing industry still faces notable challenges in its green transition. Among the three key dimensions—resource environment, energy utilization, and green investment—the resource environment exhibits relatively better performance. However, overall green development remains at a low level, particularly in energy utilization and green investment, which show significant deficiencies. The average membership degree for all three dimensions remains below 0.5, indicating that green development is still in its early stages and requires substantial improvement. Energy utilization remains a critical bottleneck, as many enterprises continue to rely on fossil fuels, and the adoption of clean energy sources has been relatively slow. Inefficient energy utilization and outdated industrial structures further hinder carbon emission reduction. To address this, the government should actively promote the adoption of energy-efficient technologies and expand the use of renewable energy through targeted policy incentives and technical support. Green investment is another pressing challenge, as financial constraints prevent many enterprises from fully embracing sustainable practices. The high initial costs of green technology adoption, coupled with inadequate incentives, discourage enterprises from making substantial investments in green development. To overcome these barriers, it is essential to strengthen green financial mechanisms, establish specialized green investment funds, and introduce preferential tax policies to attract more capital into the green economy.

### 4.2 Trends in green development quality

**Figure 7:**
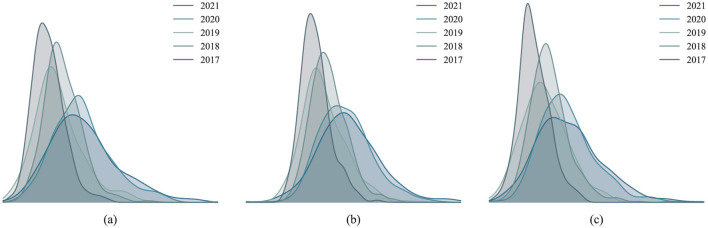
Kernel distribution status of the criterion layer. **(a)** Resources and environment. **(b)** Energy utilization. **(c)** Green investment.

To analyze the trends in the quality of green development within the manufacturing industry, we applied the Kernel analysis method to examine the evolutionary characteristics of three key dimensions: Resources and Environment, Energy Utilization, and Green Investment, as illustrated in Figure 7. This method allows us to observe distributional shifts and overall developmental trends in the green transformation of coastal manufacturing over time.

The results reveal a clear rightward shift in the distribution curves, indicating continuous improvements in the quality of green transformation within China's coastal manufacturing industry. This trend reflects steady progress in green development, largely driven by dual-carbon goals, as enterprises optimize resource utilization, enhance energy efficiency, and increase green investments. Several key factors have contributed to this progress, including stricter environmental regulations, economic restructuring toward green industries, and the widespread adoption of energy-efficient technologies. Furthermore, increased financial support for green projects, the promotion of renewable energy, and the expansion of carbon trading mechanisms have further reinforced this upward trajectory. Moreover, the peak values of the distribution curves initially rise and subsequently decline, suggesting that spatial disparities in green development quality first widened before gradually narrowing. In the early stages, these regional differences were more pronounced due to disparities in access to green technologies, variations in policy implementation, and differing industrial structures. However, over time, policy guidance, technological diffusion, and regional synergy effects have helped narrow the gap. The narrowing of disparities is closely tied to national and regional efforts, such as government incentives, the establishment of industrial green development zones, and the strengthening of enterprises' capabilities in green technological innovation. These measures have enabled underdeveloped regions to catch up, reducing the disparity between leading and lagging areas in green development quality.

Notably, all three curves exhibit right-skewed tails, indicating that while some regions have made significant progress in green development quality, others continue to lag. This regional polarization may result from differences in resource endowments, technological absorption capacities, and the implementation of local government policies. For instance, economically developed regions—supported by strong financial resources, advanced technologies, and favorable policies—have taken the lead in green transformation, whereas regions with weaker economic foundations, later development starts, or limited resource endowments have progressed more slowly. Furthermore, disparities in skilled labor availability, the pace of industrial upgrading, and market demand for green products further contribute to these regional imbalances.

Overall, while substantial progress has been made in the green transformation of China's coastal manufacturing industry, further efforts are required to promote coordinated regional development. Future strategies should focus on enhancing the diffusion and sharing of green technologies, strengthening interregional cooperation mechanisms, and establishing financial and policy support systems to assist underdeveloped regions. Encouraging technology transfer, fostering industrial clusters centered on green innovation, and improving regional policy coordination will be crucial for achieving a high-quality green transformation across the manufacturing sector. By implementing these measures, China's coastal manufacturing industry can attain more balanced and sustainable green development.

### 4.3 Regional disparity analysis

**Figure 8:**
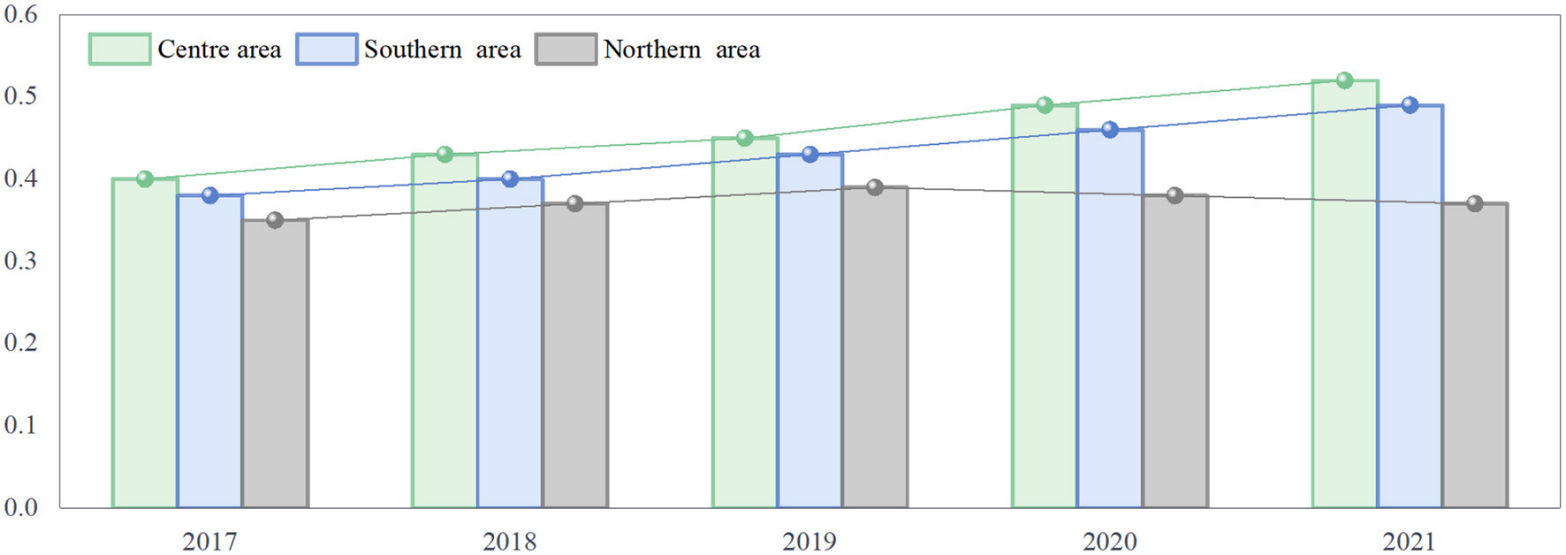
Regional differences in the green development of coastal manufacturing industries from 2017 to 2021.

To analyze the regional differences in the quality of green development in manufacturing, this paper uses the Dagum Gini coefficient method to measure the differences between the northern, southern, and central coastal regions, with the results shown in Figure 8. Overall, the Gini coefficient trends in the three regions show significant differences, forming a pattern of decreasing values from central to southern to northern regions. From the perspective of inter-regional differences, the Gini coefficients in the central and southern regions have continued to rise, indicating that the green development levels of manufacturing within these regions are becoming more polarized. Some provinces have made significant progress in green and low-carbon manufacturing, while others still face major challenges. Core provinces such as Shanghai and Guangdong have far surpassed other provinces in terms of industrial green and low-carbon development quality, thanks to industrial upgrading, green technological innovation, and strict environmental policies, thereby widening the development gap within these regions. Additionally, some cities in the southern region, such as Shenzhen and Guangzhou, continue to lead in green manufacturing, but areas like Guangxi and Hainan still need to improve in terms of investment in green development and technological application, which further exacerbates the internal regional differences. In contrast, the Gini coefficient in the northern region has been declining since 2019, indicating an imbalance in green manufacturing development. Some coastal cities, such as Dalian and Yantai, have made progress in high-end manufacturing and green industrial transformation, gradually narrowing the gap with more developed areas. However, traditional heavy industrial cities like Tangshan and Yingkou, where industries such as steel and petrochemicals account for a large proportion, have relatively slow progress in green transformation, resulting in significant internal regional disparities.

To further explore the sources of regional differences in the quality of green transformation in manufacturing, this paper further measures the intra-regional and inter-regional differences in three dimensions: resource environment, energy utilization, and green investment. The results are shown in Table 6. According to the results, the contribution rates of intra-regional differences in the three dimensions of manufacturing green development quality are 36.5%, 25.7%, and 33.3%, respectively. Among these, the intra-regional contribution of resource environment is the largest, followed by green investment, and lastly energy utilization, indicating that intra-regional differences in manufacturing green transformation mainly come from resource environment and green investment. Moreover, the contribution rates of inter-regional differences in the three dimensions are 38.3%, 37.6%, and 54.4%, respectively. Of these, green investment has the largest contribution to inter-regional differences, while the contributions of resource environment and energy utilization to inter-regional differences are relatively small. Compared to intra-regional differences, the inter-regional differences in the green development of coastal manufacturing more prominently stem from disparities in green investment.

**Table 6:** Three-dimensional regional difference contribution rate (%).

**Criterion**	**Difference**	**2017**	**2018**	**2019**	**2020**	**2021**	**Mean**
Resource environment	Within-region	36.3	35.5	37.1	36.2	37.4	36.5
	Between-region	37.2	38.5	37.1	38.9	39.7	38.3
Energy utilization	Within-region	25.0	25.5	26.7	25.1	26.3	25.7
	Between-region	36.5	35.2	38.1	39.2	39.0	37.6
Green investment	Within-region	32.2	33.1	32.4	33.7	34.9	33.3
	Between-region	53.6	52.1	55.4	56.9	54.1	54.4

## 5 Conclusion

The paper introduces a novel evaluation index system based on the AHP to support the green transformation of the manufacturing industry in China's coastal regions under the dual-carbon circular economy, thereby fostering sustainable development. The proposed system evaluates the industry's green development quality across three key dimensions: resource environment, energy utilization, and green investment. To further investigate the quality of green transformation in manufacturing, this study applies kernel analysis to examine regional evolutionary trends and the Dagum Gini coefficient method to assess regional disparities. Based on these analyses, the results indicate that the manufacturing industry must not only address technical and managerial deficiencies but also enhance the synergy between finance and policy to cultivate endogenous momentum for green development. Therefore, this study proposes the following recommendations:

1. The green transformation of the manufacturing industry is a systemic, long-term, and strategic process, making the establishment of clear development goals crucial for driving technological innovation and industrial transformation throughout the entire lifecycle of industrial products. The government should align its efforts with the dual-carbon goals while ensuring national energy security, with a particular focus on strengthening the top-level design for carbon peaking—especially in the northern coastal regions, where the foundation for green development is weak and challenges are more significant. In these regions, medium- and long-term technological roadmaps should be developed, supported by corresponding policy measures to improve the foundational capacity for carbon reduction. Prioritizing cities with favorable conditions to achieve carbon peaking first is recommended. In contrast, the central and southern coastal regions should concentrate on establishing a comprehensive statistical and accounting system to help enterprises accurately monitor their carbon emissions, providing data support for the formulation of scientifically grounded emission reduction targets and measures.
2. The government should encourage financial institutions to increase credit support for green industries. This can be achieved by establishing special funds, offering preferential interest rates, and implementing tax reductions to lower financing costs for green enterprises. At the same time, government departments should promote the creation of a green credit sharing platform for enterprises, integrating various types of green credit information to enhance financing accessibility for green businesses. Additionally, to bridge the regional gap in green development, the government should actively engage in international green finance cooperation and exchanges, aligning with advanced global standards to build a high-level green manufacturing system.
3. Enterprises in different regions should define their green transformation goals, tasks, and strategies based on local conditions and market demands. For instance, the northern coastal region can capitalize on its heavy industry clusters to prioritize the adoption of hydrogen metallurgy technology in the steel industry and carbon capture technology in the petrochemical industry, while aligning these efforts with the development of the Bohai Rim New Energy Base. The central coastal region should leverage its concentration of innovation resources to accelerate the research, development, and application of key areas such as green factories and green supply chains. Meanwhile, the southern coastal region needs to reorganize the spatial distribution of productivity and improve the green manufacturing management system to foster higher-quality green development in the manufacturing industry.
4. Coastal regions should establish regional green industry alliances to facilitate the free flow and optimal allocation of resources, such as green technologies, talent, and capital. Each region needs to leverage its unique resource endowments, industrial foundation, and development potential to strategically plan the industrial layout of the manufacturing sector. This approach will help form green industry clusters, enabling the realization of economies of scale and synergies, thus enhancing the overall competitiveness of regional manufacturing industries. In this process, the establishment of cross-regional green technology innovation platforms is essential. Regions should integrate various innovation resources and collaborate on joint efforts and innovations.

While this study provides a comprehensive evaluation of the green transformation of the manufacturing industry in China's coastal regions, several limitations remain. First, the assessment framework primarily relies on available statistical data, which may not fully capture the dynamic and qualitative dimensions of green development, such as corporate environmental responsibility and the diffusion of technological innovations. Future research could address this limitation by integrating micro-level enterprise data or employing qualitative case studies for deeper insights. Second, this study focuses predominantly on coastal regions, which are characterized by relatively advanced industrial structures and strong policy support for green development. However, inland manufacturing regions may face distinct challenges and have different policy environments. Expanding the scope of analysis to include inland areas could provide a more nuanced understanding of regional disparities in the green transformation process. Lastly, the dual-carbon circular economy, as an evolving policy framework with global implications, is subject to external factors such as global economic fluctuations, changes in energy prices, and international trade policies, all of which may influence the development of green manufacturing. Future studies should incorporate dynamic models that account for these uncertainties. Furthermore, comparative benchmarking against international standards or practices in similar coastal manufacturing regions could enhance the robustness of policy recommendations, which will be a central focus of future research.

## Data Availability

The original contributions presented in the study are included in the article/supplementary material, further inquiries can be directed to the corresponding author.
